# Supplementary material for: Recent and convergent reversion to serotype Ogawa in the AFR12 sublineage of Vibrio cholerae O1 El Tor in Cameroon
Source: Microb Genom. 2025 Sep 26;11(9):001492. doi: 10.1099/mgen.0.001492 (PMC13293293; doi:10.1099/mgen.0.001492)
Supplement: Uncited Fig. S1. [file mgen-11-01492-s001.pdf]

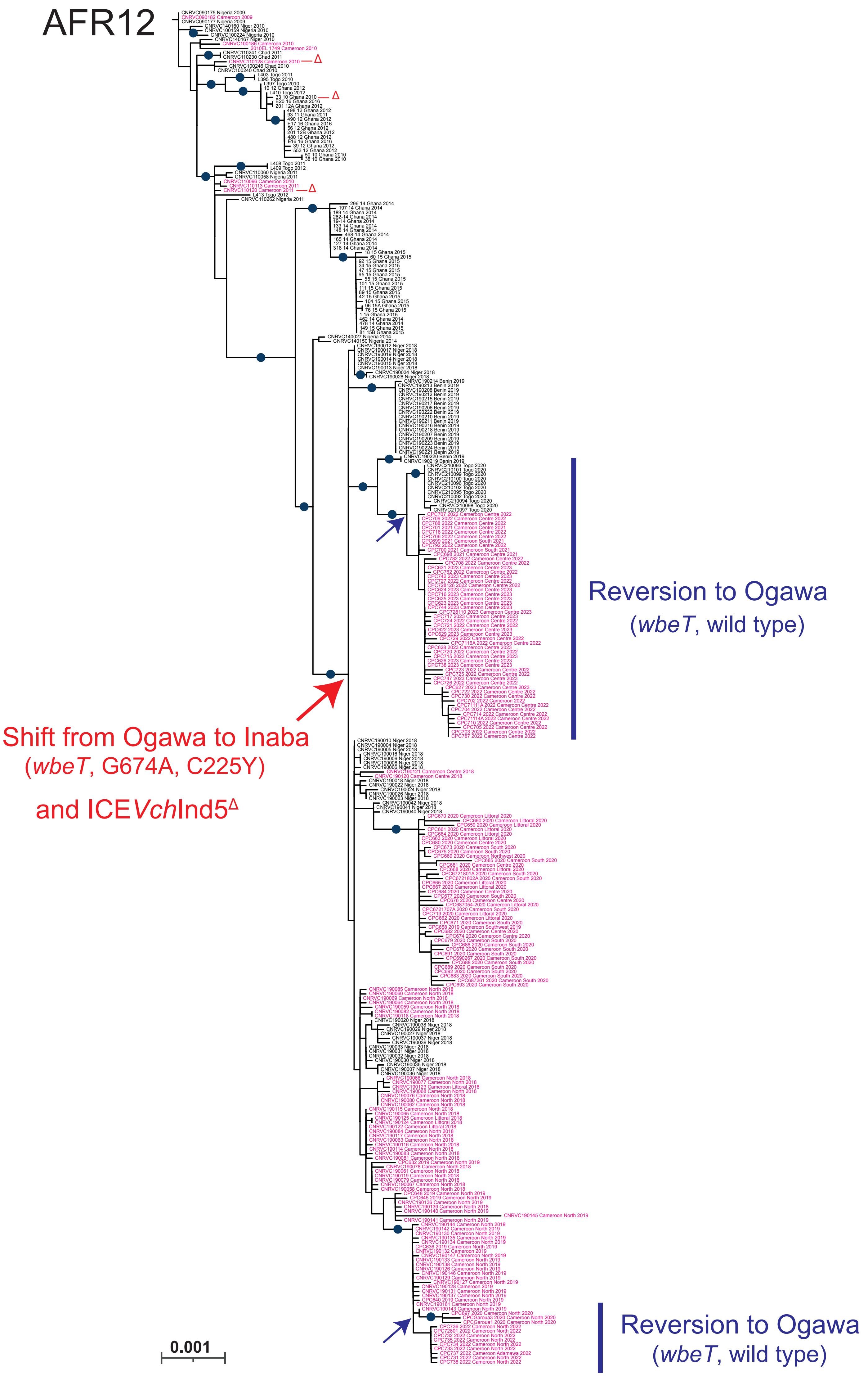
AFR12
Reversion to Ogawa
(wbeT, wild type)
Shift from Ogawa to Inaba
(wbeT, G674A, C225Y)
and ICEVchInd5Δ
Reversion to Ogawa
(wbeT, wild type)
0.001
